# *N-acetyltransferase 2* Polymorphisms and Risk of Esophageal Cancer in a Chinese Population

**DOI:** 10.1371/journal.pone.0087783

**Published:** 2014-02-19

**Authors:** Liming Wang, Weifeng Tang, Suocheng Chen, Yangyong Sun, Yu Fan, Yijun Shi, Jingfeng Zhu, Xu Wang, Liang Zheng, Aizhong Shao, Guowen Ding, Chao Liu, Ruiping Liu, Jun Yin, Haiyong Gu

**Affiliations:** 1 Cancer institute, Department of Chemotherapy, People's Hospital Affiliated to Jiangsu University, Zhenjiang, Jiangsu, China; 2 Department of Cardiothoracic Surgery, Affiliated People's Hospital of Jiangsu University, Zhenjiang, China; 3 Department of Cardiothoracic Surgery, The First People's Hospital of Changzhou and The Third Affiliated Hospital of Suzhou University, Changzhou, China; 4 Department of Orthopedics, Affiliated Hospital of Nanjing Medical University, Changzhou Second People's Hospital, Changzhou, China; Duke Cancer Institute, United States of America

## Abstract

Esophageal cancer was the fifth most commonly diagnosed cancer and the fourth leading cause of cancer-related death in China in 2009. Genetic factors might play an important role in the carcinogenesis of esophageal squamous cell carcinoma (ESCC). We conducted a hospital-based case-control study to evaluate ten *NAT2* tagging single nucleotide polymorphisms (SNPs) on the risk of ESCC. Six hundred and twenty-nine ESCC cases and 686 controls were recruited. Their genotypes were determined using the ligation detection reaction method. In the single locus analyses, there was a borderline statistically significant difference in genotype frequencies of *NAT2* rs1565684 T>C SNP between the cases and the controls (*p* = 0.057). The *NAT2* rs1565684 CC genotype was associated with a borderline significantly increased risk for ESCC (CC *vs.* TT: adjusted OR = 1.77, 95% CI = 0.97–3.21, *p* = 0.063 and CC *vs.* TT/TC: adjusted OR = 1.68, 95% CI = 0.93–3.04, *p* = 0.085). The association was evident among older patients and patients who never drunk. After the Bonferroni correction, in all comparison models, *NAT2* rs1565684 T>C SNP was not associated with ESCC risk (*p*>0.05). For the other nine *NAT2* SNPs, after Bonferroni correction, in all comparison models, the nine SNPs were also not associated with ESCC risk (*p*>0.05). Thus, nine *NAT2* tagging SNPs were not associated with risk of ESCC. *NAT2* rs1565684 T>C SNP might play a slight role in ESCC etiology. Additional, larger studies and tissue-specific biological characterization are required to confirm the current findings.

## Introduction

Esophageal cancer was the fourth leading cause of cancer death and the fifth most commonly diagnosed cancer in China in 2009 [1]. Genetic factors, such as single nucleotide polymorphisms (SNPs), might play an important role in the carcinogenesis of esophageal squamous cell carcinoma (ESCC) [2].

N-acetyltransferase 2 (NAT2) is an enzyme that plays an essential role in the metabolism of various potential carcinogens. NAT2 is mainly expressed in the human liver and gastrointestinal tract. The *NAT2* gene is located on 8p21.3-23.1 and encodes a 290-amino acid protein, NAT2 [3]. *NAT2* is polymorphic, and it was thought that NAT2 acetylation status alteration caused by *NAT* polymorphisms decreased enzymatic activity and result in absence of detoxification efficiency, which could lead to an increase in cancer susceptibility [4]. It has been reported that *NAT2* polymorphisms and/or their interaction with smoking is associated with various types of malignancies.

Genetic variation of *NAT2* may lead to differences in the rate of arylamine metabolism and consequently increase cancer risk [5]. The substrates for NAT2 that are involved in carcinogenesis, are represented mainly by heterocyclic amines and polycyclic aromatic hydrocarbon rings found in cooked or smoked meat [6] and cigarette smoke [7].

*NAT2* genetic variations may contribute to the development of ESCC. In a hospital-based case-control study, we performed genotyping analyses of ten *NAT2* tagging SNPs in 629 ESCC cases and 686 controls in a Chinese population.

## Materials and Methods

### Ethical approval of the study protocol

The Review Board of Jiangsu University (Zhenjiang, China) approved this hospital-based case-control study. We have complied with the World Medical Association Declaration of Helsinki regarding ethical conduct of research involving human subjects and/or animals. All subjects provided written, informed consent to be included in the study.

### Patients and Controls

Six hundred and twenty-nine subjects with esophageal cancer were consecutively recruited from the Affiliated People's Hospital of Jiangsu University and Affiliated Hospital of Jiangsu University (Zhenjiang, China) between October 2008 and December 2010. All cases of esophageal cancer were diagnosed as ESCC pathologically. The exclusion criteria were patients who had previously had: cancer; any metastasized cancer; radiotherapy or chemotherapy. The 686 controls were patients without cancer and were matched to the cases with regard to age (±5 years) and sex. They were recruited from the two hospitals mentioned above during the same time period. Most of the controls were admitted to the hospitals for the treatment of trauma.

Trained interviewers, using a pre-tested questionnaire, questioned each subject personally to obtain information on demographic data (e.g., age, sex) and related risk factors (including tobacco smoking and alcohol consumption). After the interview, 2-mL samples of venous blood were collected from each subject. Individuals who smoked one cigarette per day for >1 year were defined as “smokers”. Subjects who consumed more than three alcoholic drinks a week for >6 months were considered to be “alcohol drinkers”.

### Isolation of DNA, SNPs selection and genotyping by ligation detection reaction

Blood samples were collected from patients using Vacutainers and transferred to tubes lined with ethylenediamine tetra-acetic acid (EDTA). Genomic DNA was isolated from whole blood with the QIAamp DNA Blood Mini Kit (Qiagen, Berlin, Germany) [8]. We used a block-based tagging strategy to find tagging SNPs using Haploview 4.2 software, according to the HapMap database (http://www.hapmap.org/, phase II Nov08, on NCBI B36 assembly, dbSNP b126; population: Chinese Han population); minor allele frequency (MAF)≥0.05, Hardy-Weinberg equilibrium (HWE) *p*≥0.05 and call rate ≥95%) on the basis of pairwise linkage disequilibrium r^2^ threshold of 0.8. Ten *NAT2* tagging SNPs were thus selected. The samples were genotyped using the ligation detection reaction (LDR) method, with technical support from the Shanghai Biowing Applied Biotechnology Company [9], [10]. For quality control, repeated analyses were done for 160 (12.17%) randomly selected samples with high DNA quality.

### Statistical Analyses

Differences in the distributions of demographic characteristics, selected variables, and genotypes of the *NAT2* variants between the cases and controls were evaluated using the *χ* ^2^ test. The associations between the ten SNPs and risk of ESCC were estimated by computing the odds ratios (ORs) and their 95% confidence intervals (CIs) using logistic regression analyses for crude ORs and adjusted ORs when adjusting for age, sex, smoking and drinking status. The Bonferroni correction procedure was applied because of the number of comparisons. The HWE was tested by a goodness-of-fit *χ* ^2^ test to compare the observed genotype frequencies to the expected frequencies among the control subjects. All statistical analyses were performed with SAS 9.1.3 (SAS Institute, Cary, NC, USA).

## Results

### Characteristics of the study population

Characteristics of cases and controls included in the study are summarized in Table 1. The cases and controls appeared to be adequately matched on age and sex as suggested by the *χ* ^2^ tests. As shown in Table 1, significant difference was detected on smoking status between the cases and the controls, and drinking rate was higher in ESCC patients than in control subjects. The primary information for eight genotyped SNPs was in Table 2. The concordance rates of repeated analyses were 100% except *NAT2* rs11996129 T>C (157/160, 98.13%), rs1565684 T>C (159/160, 99.38%) and rs1799930 G>A (159/160, 99.38%). MAF in our controls was similar to MAF for Chinese in database for all SNPs. The observed genotype frequencies for these ten polymorphisms in the controls were all in HWE except *NAT2* rs4540438 A>C (*p* = 0.015) (Table 2).

**Table 1:** Distribution of selected demographic variables and risk factors in ESCC cases and controls.

Variable	Cases (n = 629)		Controls (n = 686)		*p* a
	n	%	n	%	
**Age (years)** mean ± SD	62.85 (±8.13)		62.58 (±7.89)		0.541
**Age (years)**					0.155
<63	310	49.28	365	53.21	
≥63	319	50.72	321	46.79	
**Sex**					0.185
Male	444	70.59	461	67.20	
Female	185	29.41	225	32.80	
**Tobacco use**					**<0.001**
Never	355	56.44	499	72.74	
Ever	274	43.56	187	27.26	
**Alcohol use**					**<0.001**
Never	428	68.04	526	76.68	
Ever	201	31.96	160	23.32	

a Two-sided *χ* ^2^ test and student t test; Bold values are statistically significant (*p*<0.05).

**Table 2:** Primary information for *NAT2* rs1041983 C>T, rs11780884 A>G, rs11996129 T>C, rs12674710 C>A, rs1390359 C>A, rs1390360 G>A, rs1565684 T>C, rs1799930 G>A, rs1799931 G>A and rs4540438 A>C polymorphisms.

Genotyped SNPs	*NAT2* rs1041983 C>T	*NAT2* rs11780884 A>G	*NAT2* rs11996129 T>C	*NAT2* rs12674710 C>A	*NAT2* rs1390359 C>A	*NAT2* rs1390360 G>A	*NAT2* rs1565684T>C	*NAT2* rs1799930 G>A	*NAT2* rs1799931 G>A	*NAT2* rs4540438 A>C
Chromosome	8	8	8	8	8	8	8	8	8	8
Gene Official Symbol	NAT2	NAT2	NAT2	NAT2	NAT2	NAT2	NAT2	NAT2	NAT2	NAT2
Function	cds-synon	No Data	intron region	No Data	No Data	No Data	No Data	missense	missense	No Data
Chr Pos (Genome Build 36.3)	18302075	18290333	18298855	18307943	18305609	18305773	18290944	18302383	18302650	18307883
Regulome DB Score^a^	5	No Data	No Data	No Data	No Data	No Data	4	6	No Data	No Data
TFBS^b^	—	Y	—	—	—	—	Y	—	—	—
Splicing (ESE or ESS)	—	—	—	—	—	—	—	—	—	—
miRNA (miRanda)	—	—	—	—	—	—	—	—	—	—
miRNA (Sanger)	—	—	—	—	—	—	—	—	—	—
nsSNP	—	—	—	—	—	—	—	Y	Y	—
MAF^c^ for Chinese in database	0.366	0.453	0.276	0.471	0.163	0.244	0.188	0.207	0.159	0.058
MAF in our controls (n = 686)	0.387	0.452	0.249	0.460	0.188	0.257	0.193	0.227	0.153	0.067
*p* value for HWE^d^ test in our controls	0.432	0.317	0.073	0.544	0.841	0.484	0.124	0.328	0.201	**0.015**
Genotyping method^e^	LDR	LDR	LDR	LDR	LDR	LDR	LDR	LDR	LDR	LDR
% Genotyping value	98.48%	96.43%	96.35%	98.18%	96.43%	98.48%	98.18%	96.81%	95.29%	98.18%

a http://www.regulomedb.org/;

b TFBS: Transcription Factor Binding Site (http://snpinfo.niehs.nih.gov/snpinfo/snpfunc.htm);

c MAF: minor allele frequency, *NAT2* rs4540438 A>C MAF is in CHB+JPT population;

d HWE: Hardy–Weinberg equilibrium;

e LDR: ligation detection reaction; Bold values are statistically significant (*p*<0.05).

### Associations between NAT2 tagging polymorphisms and risk of ESCC

The genotype distributions of *NAT2* rs1565684 T>C in the cases and the controls are shown in Table 3. In the single locus analyses, there was a borderline statistically significant difference in genotype frequencies of *NAT2* rs1565684 T>C SNP between the cases and the controls (*p* = 0.057). When the *NAT2* rs1565684 TT homozygote genotype was used as the reference group, the TC genotype was not associated with the risk for ESCC (TC vs. TT: OR = 1.14, 95% CI = 0.90–1.44, *p* = 0.269); the CC genotype was associated with a significantly increased risk for ESCC (CC vs. TT: OR = 1.95, 95% CI = 1.08–3.51, *p* = 0.026). In the dominant model, the *NAT2* rs1565684 TC/CC variants were not associated with the risk of ESCC, compared with the *NAT2* rs1565684 TT genotype (TC/CC vs. TT: OR = 1.20, 95% CI = 0.96–1.51, *p* = 0.107). In the recessive model, when the *NAT2* rs1565684 TT/TC genotypes were used as the reference group, the CC homozygote genotype was associated with an 86% increased risk of ESCC (CC vs. TT/TC: OR = 1.86, 95% CI = 1.04–3.33, *p* = 0.037) (Table 3). After adjusted for age, sex, smoking and drinking status, the CC genotype was associated with a borderline significantly increased risk for ESCC (CC vs. TT: adjusted OR = 1.77, 95% CI = 0.97–3.21, *p* = 0.063 and CC vs. TT/TC: adjusted OR = 1.68, 95% CI = 0.93–3.04, *p* = 0.085). After the Bonferroni correction, in all comparison models, *NAT2* rs1565684 T>C SNP was not associated with ESCC risk (*p*>0.05).

**Table 3:** Logistic regression analyses of associations between *NAT2* rs1041983 C>T, rs11780884 A>G, rs11996129 T>C, rs12674710 C>A, rs1390359 C>A, rs1390360 G>A, rs1565684 T>C, rs1799930 G>A, rs1799931 G>A and rs4540438 A>C polymorphisms and risk of ESCC.

Genotype	Cases (n = 629)		Controls (n = 686)		Crude OR (95%CI)	*p*	Adjusted OR^a^ (95%CI)	*p*
	n	%	n	%				
*NAT2* rs1041983 C>T								
CC	209	33.8	259	38.3	1.00		1.00	
CT	315	50.9	311	46.0	1.26 (0.99–1.60)	0.064	1.23 (0.96–1.57)	0.097
TT	95	15.3	106	15.7	1.11 (0.80–1.55)	0.535	1.13 (0.81–1.59)	0.478
CT+TT	410	66.2	417	61.7	1.22 (0.97–1.53)	0.089	1.21 (0.96–1.52)	0.114
CC+CT	524	84.7	570	84.3	1.00		1.00	
TT	95	15.3	106	15.7	0.98 (0.72–1.32)	0.869	1.00 (0.74–1.37)	0.979
*NAT2* rs11780884 A>G								
AA	165	27.3	206	31.0	1.00		1.00	
AG	303	50.2	316	47.6	1.20 (0.93–1.55)	0.172	1.18 (0.91–1.53)	0.219
GG	136	22.5	142	21.4	1.20 (0.88–1.63)	0.261	1.22 (0.89–1.68)	0.215
AG+GG	439	72.7	458	69.0	1.20 (0.94–1.53)	0.148	1.19 (0.93–1.53)	0.163
AA+AG	468	77.5	522	78.6	1.00		1.00	
GG	136	22.5	142	21.4	1.07 (0.82–1.39)	0.627	1.10 (0.84–1.45)	0.479
*NAT2* rs11996129 T>C								
TT	345	56.2	377	57.7	1.00		1.00	
TC	236	38.4	227	34.8	1.14 (0.90–1.44)	0.284	1.16 (0.92–1.47)	0.220
CC	33	5.4	49	7.5	0.74 (0.46–1.17)	0.196	0.80 (0.50–1.29)	0.366
TC+CC	269	43.8	276	42.3	1.07 (0.85–1.33)	0.579	1.10 (0.88–1.38)	0.411
TT+TC	581	94.6	604	92.5	1.00		1.00	
CC	33	5.4	49	7.5	0.70 (0.44–1.10)	0.125	0.76 (0.47–1.21)	0.243
*NAT2* rs12674710 C>A								
CC	166	27.2	202	29.7	1.00		1.00	
CA	309	50.6	330	48.5	1.14 (0.88–1.47)	0.321	1.11 (0.86–1.45)	0.421
AA	136	22.3	148	21.8	1.19 (0.82–1.53)	0.482	1.15 (0.84–1.58)	0.388
CA+AA	445	72.8	478	70.3	1.13 (0.89–1.44)	0.314	1.13 (0.88–1.44)	0.351
CC+CA	475	77.7	532	78.2	1.00		1.00	
AA	136	22.3	148	21.8	1.03 (0.79–1.34)	0.831	1.07 (0.82–1.41)	0.604
*NAT2* rs1390359 C>A								
CC	412	67.4	434	66.1	1.00		1.00	
CA	179	29.3	199	30.3	0.95 (0.74–1.21)	0.664	0.96 (0.75–1.23)	0.735
AA	20	3.3	24	3.7	0.88 (0.48–1.61)	0.676	1.03 (0.55–1.91)	0.930
CA+AA	199	32.6	223	33.9	0.94 (0.74–1.19)	0.605	0.97 (0.76–1.23)	0.771
CC+CA	591	96.7	633	96.3	1.00		1.00	
AA	20	3.3	24	3.7	0.89 (0.49–1.63)	0.713	1.04 (0.56–1.93)	0.896
*NAT2* rs1390360 G>A								
GG	343	55.4	377	55.8	1.00		1.00	
GA	242	39.1	251	37.1	1.06 (0.84–1.33)	0.620	1.07 (0.85–1.36)	0.555
AA	34	5.5	48	7.1	0.78 (0.49–1.24)	0.289	0.88 (0.55–1.42)	0.609
GA+AA	276	44.6	299	44.2	1.02 (0.82–1.26)	0.897	1.05 (0.84–1.31)	0.703
GG+GA	585	94.5	628	92.9	1.00		1.00	
AA	34	5.5	48	7.1	0.76 (0.48–1.20)	0.237	0.86 (0.54–1.37)	0.519
*NAT2* rs1565684 T>C								
TT	366	59.9	437	64.3	1.00		1.00	
TC	214	35.0	224	32.9	1.14 (0.90–1.44)	0.269	1.14 (0.90–1.45)	0.270
CC	31	5.1	19	2.8	**1.95 (1.08–3.51)**	**0.026**	1.77 (0.97–3.21)	0.063
TC+CC	245	40.1	243	35.7	1.20 (0.96–1.51)	0.107	1.19 (0.95–1.50)	0.130
TT+TC	580	94.9	661	97.2	1.00		1.00	
CC	31	5.1	19	2.8	**1.86 (1.04–3.33)**	**0.037**	1.68 (0.93–3.04)	0.085
*NAT2* rs1799930 G>A								
GG	375	62.5	407	60.5	1.00		1.00	
GA	200	33.3	227	33.7	0.96 (0.76–1.21)	0.711	0.97 (0.76–1.23)	0.804
AA	25	4.2	39	5.8	0.70 (0.41–1.17)	0.173	0.78 (0.45–1.33)	0.353
GA+AA	225	37.5	266	39.5	0.92 (0.73–1.15)	0.459	0.94 (0.75–1.19)	0.620
GG+GA	575	95.8	634	94.2	1.00		1.00	
AA	25	4.2	39	5.8	0.71 (0.42–1.18)	0.186	0.78 (0.46–1.33)	0.367
*NAT2* rs1799931 G>A								
GG	406	67.3	462	71.1	1.00		1.00	
GA	181	30.0	177	27.2	1.16 (0.91–1.49)	0.228	1.15 (0.89–1.47)	0.289
AA	16	2.7	11	1.7	1.66 (0.76–3.61)	0.205	1.35 (0.61–2.99)	0.465
GA+AA	197	32.7	188	28.9	1.19 (0.94–1.52)	0.151	1.16 (0.91–1.48)	0.240
GG+GA	587	97.3	639	98.3	1.00		1.00	
AA	16	2.7	11	1.7	1.58 (0.73–3.44)	0.246	1.29 (0.59–2.86)	0.526
*NAT2* rs4540438 A>C								
AA	522	85.4	596	87.6	1.00		1.00	
AC	86	14.1	77	11.3	1.28 (0.92–1.77)	0.148	1.31 (0.93–1.83)	0.118
CC	3	0.5	7	1.0	0.49 (0.13–1.90)	0.302	0.52 (0.13–2.04)	0.344
AC+CC	89	14.6	84	12.4	1.21 (0.88–1.67)	0.243	1.24 (0.90–1.72)	0.195
AA+AC	608	99.5	673	99.0	1.00		1.00	
CC	3	0.5	7	1.0	0.48 (0.12–1.84)	0.282	0.50 (0.13–1.97)	0.319

a Adjusted for age, sex, smoking status and alcohol consumption; Bonferroni correction was performed to correct the *p* value (*p*_correct_); For the 10 *NAT2* SNPs, *p*_correct_>0.05 in all comparison models; Bold values are statistically significant (*p*<0.05).

For the other nine SNPs, in the single locus analyses, there was no statistically significant difference in genotype frequencies of these nine SNPs between the cases and the controls (*p*>0.05). Logistic regression analyses revealed that none of these nine polymorphic sites was associated with the susceptibility to ESCC. In all comparison models, the nine SNPs were not associated with ESCC risk (*p*>0.05) before and after the Bonferroni correction (Table 3).

### Stratification analyses of NAT2 rs1565684 T>C polymorphisms and risk of ESCC

To evaluate the effects of *NAT2* rs1565684 T>C genotypes on ESCC risk according to different age, sex, smoking and alcohol drinking status; we performed the stratification analyses (Table 4). A significantly increased risk of ESCC associated with the *NAT2* rs1565684 T>C polymorphism was evident among older patients and patients who never drunk (Table 4).

**Table 4:** Stratified analyses between *NAT2* rs1565684 T>C polymorphism and ESCC risk by sex, age, smoking status and alcohol consumption.

Variable	*NAT2* rs1565684 T>C (case/control)^a^				Adjusted OR^b^ (95% CI); *p*; *p*_h_ c				
	TT	TC	CC	TC+CC	TT	TC	CC	TC+CC	CC vs. (TC+TT)
Sex									
Male	263/290	143/151	26/14	169/165	1.00	1.04 (0.78–1.38); *p*: 0.815; *p*_h_:0.265	1.86 (0.94–3.68); *p*: 0.077; *p*_h_:0.621	1.11 (0.84–1.46); *p*: 0.472; *p*_h_:0.402	1.83 (0.93–3.61); *p*: 0.080; *p*_h_:0.519
Female	103/147	71/73	5/5	76/78	1.00	1.41 (0.93–2.13); *p*: 0.106; *p*_h_:0.265	1.53 (0.43–5.45); *p*: 0.516; *p*_h_:0.621	1.42 (0.94–2.13); *p*: 0.094; *p*_h_:0.402	1.34 (0.38–4.75); *p*: 0.648; *p*_h_:0.519
Age									
<63	187/231	97/124	15/8	112/132	1.00	0.92 (0.66–1.30); *p*: 0.645; *p*_h_:0.165	1.73 (0.70–4.31); *p*: 0.239; *p*_h_:0.591	0.98 (0.70–1.36); *p*: 0.891; *p*_h_:0.234	1.78 (0.72–4.39); *p*: 0.211; *p*_h_:0.458
≥63	179/206	117/100	16/11	133/111	1.00	1.38 (0.99–1.94); *p*: 0.059; *p*_h_:0.165	1.73 (0.78–3.87); *p*: 0.178; *p*_h_:0.591	**1.42 (1.02–1.96); ** ***p*** **: 0.035;** *p*_h_:0.234	1.54 (0.70–3.41); *p*: 0.284; *p*_h_:0.458
Smoking status									
Never	205/323	125/160	14/11	139/171	1.00	1.22 (0.91–1.64); *p*: 0.190; *p*_h_:0.378	2.03 (0.90–4.59); *p*: 0.088; *p*_h_:0.636	1.27 (0.95–1.70); *p*: 0.102; *p*_h_:0.398	1.89 (0.84–4.25); *p*: 0.122; *p*_h_:0.729
Ever	161/114	89/64	17/8	106/72	1.00	1.05 (0.70–1.58); *p*: 0.821; *p*_h_:0.378	1.51 (0.62–3.69); *p*: 0.368; *p*_h_:0.636	1.10 (0.74–1.63); *p*: 0.634; *p*_h_:0.398	1.48 (0.61–3.58); *p*: 0.382; *p*_h_:0.729
Alcohol consumption									
Never	252/341	142/169	21/11	163/180	1.00	1.17 (0.88–1.56); *p*: 0.273; *p*_h_:0.908	**2.38 (1.10–5.14); ** ***p*** **: 0.028;** *p*_h_:0.150	1.25 (0.95–1.65); *p*: 0.114; *p*_h_:0.664	**2.25 (1.05–4.83); ** ***p*** **: 0.038;** *p*_h_:0.149
Ever	114/96	72/55	10/8	82/63	1.00	1.12 (0.71–1.77); *p*: 0.615; *p*_h_:0.908	1.14 (0.43–3.02); *p*: 0.799; *p*_h_:0.150	1.13 (0.73–1.74); *p*: 0.596; *p*_h_:0.664	1.09 (0.42–2.85); *p*: 0.866; *p*_h_:0.149

a The genotyping was successful in 611 (97.1%) ESCC cases, and 680 (99.1%) controls for *NAT2* rs1565684 T>C;

b Adjusted for age, sex, smoking status and alcohol consumption (besides stratified factors accordingly) in a logistic regression model;

c *p*_h_ for heterogeneity.

## Discussion

In this hospital-based case-control study of ESCC, we found that ten selected *NAT2* tagging SNPs were not associated with the risk of ESCC after the Bonferroni correction. *NAT2* rs1565684 CC genotype was associated with a borderline significantly increased risk for ESCC. A significantly increased risk of ESCC associated with the *NAT2* rs1565684 T>C polymorphism was evident among older patients and patients who never drunk. To the best of our knowledge, it's the first positive finding of *NAT2* rs1565684 T>C polymorphism and ESCC risk.

NAT2 is involved in the metabolism of a major class of tobacco smoke carcinogens (the aromatic amines) and *NAT2* variant alleles result in slow clearance of aromatic amines. In humans, the *NAT2* gene encodes a phase II enzyme that plays an essential role in aromatic, heterocyclic amines and hydrazines metabolism [11]. NAT2 influences the detoxification of aromatic and heterocyclic amine carcinogens (which are present in tobacco smoke) by two pathways: the metabolism reaction may result in the detoxification by N-acetylation, or bioactivation by O-acetylation often preceded by CYP450 hydroxylation [11].

Previous case-control reports have yielded inconsistent results regarding the association of *NAT2* SNPs with cancers, possibly because of the small number of subjects, which would compromise the power of the statistical analyses in these studies. In the esophagus, the slow *NAT2* acetylator genotype was more susceptible to esophageal cancer in Japan [12]. However, in another study in Taiwan, *NAT2* polymorphisms did not affect the risk of esophageal cancer, irrespective of environmental factors [13]. In a more recent study in India, *NAT2* acetylator genotypes did not influence susceptibility to esophageal cancer. *NAT2* polymorphisms did not significantly modulate the cancer risk after interaction with environmental factors, such as tobacco, alcohol or occupational exposure [14]. In another study in the Kashmir Valley, none of the three *NAT2* polymorphic alleles (rs1799929, rs1799930 and rs1799931) was found to be independently associated with risk of esophageal and gastric cancers [15], which was also in accordance with our results. Meta analysis also suggested that *NAT2* genotypes are not associated with lung cancer [16], gastric cancer [17], breast cancer [18], prostate cancer [19] and oral cancer [20]. *NAT2* rs1565684 T>C is in linkage disequilibrium with another important SNP *NAT2* rs4345600 A>G (NS 12, −9306 A>G) (r^2^ = 0.845) in Chinese Han Beijing population. Although *NAT2* rs1565684 T>C SNP is functional using SNP function prediction websites (http://snpinfo.niehs.nih.gov/snpinfo/snpfunc.htm and http://www.regulomedb.org/). The etiology of *NAT2* rs1565684 T>C SNP is still not well known and need further investigation.

This case-control study had several limitations. First, the patients and controls were enrolled from hospitals; inherent bias may have resulted in spurious findings. Second, the statistical power of our study was limited because of the moderate sample size and absence of a validation cohort; further replication studies are needed. Third, the viral infections and immune parameters information were not available, which restricted the power of our analyses. Finally, we did not obtain detailed information on cancer metastasis and survival, which restricted further analyses of the roles of the *NAT2* polymorphisms in ESCC progression and prognosis.

In conclusion, our study provides evidence that *NAT2* tagging SNPs may not contribute to the risk of ESCC. Larger well-designed studies are required to confirm the current findings.
